# Dissociative Symptoms and Mother's Marital Status in Young Adult Population

**DOI:** 10.1097/MD.0000000000000408

**Published:** 2015-01-16

**Authors:** Petr Bob, Petra Selesova, Jiri Raboch, Lubomir Kukla

**Affiliations:** From the Department of Psychiatry & UHSL, 1st Faculty of Medicine, Center for Neuropsychiatric Research of Traumatic Stress, Charles University, Prague (PB, PS, JR); RECETOX, Faculty of Science (LK); CEITEC, Medical Faculty, Masaryk University, Brno, Czech Republic (PB).

## Abstract

Current findings suggest that mother's marital status indicating father's absence or conflicting relationship to father may be specifically related to dissociation and other stress-related symptoms.

We have assessed relationships of mother's marital status, dissociative symptoms, and other psychopathological manifestations in a sample of 19 years’ old young adults (N = 364) participating in European longitudinal study (European Longitudinal Study of Parenthood and Childhood).

The results show clinically significant manifestations of dissociative symptoms in young adult men whose mothers were fatherless and in women whose mothers were re-married. Other psychopathological symptoms did not reach clinically significant manifestations.

The results suggest that significant factor related to high level of dissociative symptoms in men growing in fatherless families might be linked with disturbed and conflicting attachment to a father's figure and pathological dependent attachment to mother. In women dissociative symptoms likely are linked to conflicting relationship between mother and daughter associated with stepfather’ presence in the family.

## INTRODUCTION

Recent empirical data indicate that relationships between parental divorce and children's emotional and behavioral problems are predominantly associated with psychological factors and there is a relationship between fatherlessness and children's emotional and behavioral problems.^1,2^ According to these findings on influence of fatherlessness, there is growing evidence that a level of father's absence in divorced families and in unmarried women who live with their child without father's person significantly influences mental health of the growing child.^2–4^ The influence of parental separation on child's mental health depends on age of the child when the separation happened. Other factors influencing child's mental health related to parental separation represent parental relationships after divorce and quality of contacts that children may have with a parent living out of the family. In this context, there is evidence that divorce and destructive couple conflict represent major risk factors for many forms of dysfunction and psychopathological manifestations in children.^5^ In addition, there is evidence that children from single parent or blended families have increased vulnerability to traumatic and other stressful life events.^6–8^

Although numerous findings show that conflicting attachment and disrupted family structure play a significant role in child psychopathology, there is no evidence indicating a relationship between disrupted family structure and dissociative symptoms that frequently occur as a response to traumatic or stressful experiences. Together these findings on stress influences related to conflicting attachment suggest a hypothesis that mother's marital status indicating father's absence in a family is related to dissociative psychopathology and other stress-related manifestations. With the aim to test the hypothesis, we have assessed whether mother's marital status will be related to dissociative symptoms, symptoms of traumatic stress, anxiety and depression, in a sample of young adults participating in the European longitudinal study (European Longitudinal Study of Parenthood and Childhood [ELSPAC]) studying parental and environmental influences on children development.

## MATERIALS AND METHODS

### Participants

Within the framework of European Longitudinal Study of Parenthood and Childhood (ELSPAC) data of 364 Czech young adults who within period of 2 months responded to invitation for participation in the study were collected. All participants included in the study were 19 years’ old, born in 1992 in the interval of 6 months with a high school education (mean age 18.5 years, age range within 1 year, more than 18 less than 19; 151 men and 213 women). All participants lived in the city of Brno, with similar social and economical status. The ELSPAC study started in 1992 in few European countries and its organization centers were in the United Kingdom and Czech Republic. The participants included in the study were selected randomly from the population based on voluntary agreement provided by parents awaiting newborn children.

To find potential mental health risks related to stress influences associated with mother's marital status, we have assessed dissociative symptoms, symptoms of traumatic stress, depression, and anxiety. All the participants gave written informed consent and the research based on collaboration of ELSPAC and Center for Neuropsychiatric Research of Traumatic Stress was approved by Charles University (First Faculty of Medicine) ethical committee.

### Psychometric Measures

Symptoms of dissociation were assessed using the Dissociative Experiences Scale (DES).^9,10^ The DES is a self-reported scale with 28 items, asking respondents to indicate their response on 100-mm scale to what extent they experience typical dissociative phenomena in daily life (Cronbach's alpha 0.92, test–retest reliability after week 0.91). Normative score of the scale defines increased probability for manifestations of dissociative disorders for total scores >30. Dissociative phenomena for example include feelings of depersonalization, derealization, psychogenic amnesia, and others. DES as well as other psychometric measures used in this study were translated into Czech language from the English original and then back-translated into English. The resulting documents were compared with the originals by a native English speaker and all the tests have good psychometric properties and equivalent quality to test occurrence of the symptoms as their English originals.

Somatoform dissociative symptoms were measured using 20-items self-reported Somatoform Dissociation Questionnaire (SDQ-20).^11,12^ Normative score of the scale defines significant occurrence of somatoform dissociative symptoms for scores >30. Somatoform dissociative symptoms represent alterations in sensations of pain (analgesia, kinesthetic anesthesia), alterations of perception, loss of motor control, gastrointestinal symptoms, etc. Subjects indicate the degree of their experience on 5-point Likert scale (Cronbach's alpha 0.91, test–retest reliability after week 0.90).

For investigation of childhood traumas, the Trauma Symptom Checklist (TSC-40)^13^ was used. The TSC-40 is a self-reported 40-item questionnaire rated on a 4-point Likert scale. TSC-40 evaluates symptomatology in adults associated with childhood or adult traumatic experiences and measures aspects of posttraumatic stress and other symptom clusters found in some traumatized individuals (Cronbach's alpha 0.91, test–retest reliability after week 0.88). Normative score of the scale assumes likely occurrence of traumatic events for scores >70.^13^

For the assessment of depressive symptoms, the Beck Depression Inventory (BDI-II)^14^ was used. The BDI-II represents 21-items questionnaire for assessing depression (Cronbach's alpha 0.89, test–retest reliability after week 0.85). Subjects indicate degree of their experience of depressive symptoms on a 4-point Likert scale. Normative score of the scale defines significant level of depression for scores >30.^14^

Levels of anxiety symptoms were assessed using the Zung's Self-Rating Anxiety Scale (SAS) (Cronbach's alpha 0.89, test–retest reliability after week 0.85).^15^ The SAS is a 20-item self-reporting questionnaire focused on the most common general anxiety symptoms. Each question is scored on 4-point Likert scale from 1 to 4. Normative score of the scale defines significant level of anxiety for scores >30.^15^

### Statistical Methods

Statistical evaluations of psychometric measures included means, standard deviations, Kruskal–Wallis analysis of variance (ANOVA), Mann–Whitney test for independent samples, and power analysis. All the methods of statistical evaluation were performed using the software package Statistica, version 6.

## RESULTS

The group of participants (N = 364) was divided into 3 subgroups according to the mother's marital status. The first Fatherless subgroup contained 60 participants and included 20 men whose mother was divorced (N = 16) or unmarried (N = 4), and 40 women whose mother was divorced (N = 36) or unmarried (N = 4). The second subgroup contained 273 participants whose mother was married and included 114 men and 159 women. The third group contained 31 participants whose mother was re-married and included 17 men and 14 women.

In the statistical analysis, all differences between these 3 subgroups using Kruskal–Wallis ANOVA for the whole group including women and men (Table 1) and separately for the men's group (Table 1) and for the women's group (Table 1) were found statistically significant for dissociative symptoms (DES at *P* < 0.0002, *z* > 4.23 and SDQ-20 at *P* < 0.002, *z* > 3.69), symptoms of traumatic stress (TSC-40 at *P* < 0.004, *z* > 3.57), depression (BDI-II at *P *< 0.005, *z* > 3.47), and anxiety (SAS at *P* < 0.005, *z* > 3.51).

**Table 1:**
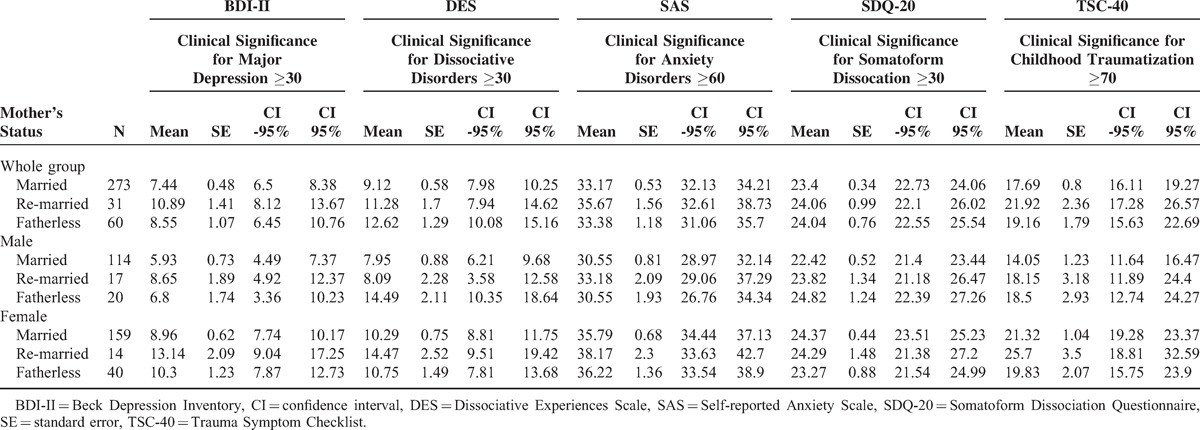
Psychometric Scores (Mean, Standard Error, Confidence Interval) for the Groups According to the Mother's Marital Status

The statistical analysis shows significant clinical values of psychological dissociative symptoms measured by DES (Table 1). All differences in DES scores between the subgroups using Kruskal–Wallis ANOVA were found statistically significant in men (*P* < 0.002, *z* > 0.76) and women (*P* < 0.0003, *z* > 4.17).

Because Kruskal–Wallis ANOVA has not well established and standardized methods of power analysis, we have estimated standardized effect sizes for the mean values characterizing differences of dissociative symptoms measured by DES between the subgroups of men and women (Table 1) that were found statistically significant with medium or high effect sizes, that is, effect size for DES means in men between Fatherless and Married subgroups is 0.75, *P* < 0.01; between Fatherless and Re-married subgroup is 0.74, *P* < 0.01; whereas the effect size between Married and Re-married subgroup was not statistically significant (population SD = 8.71). The effect size for DES Means in women between Remarried and Married subgroup is 0.45, *P* < 0.01; between Remarried and Fatherless subgroup is 0.43, *P* < 0.01; whereas the effect size between Married and Fatherless subgroup is not significant.

The results also indicate that psychological dissociative symptoms measured by DES are significantly correlated to symptoms of somatoform dissociation (SDQ-20) (Spearman r = 0.55, *P* < 0.01), traumatic stress (TSC-40) (Spearman r = 0.52, *P* < 0.01), depression (BDI-II) (Spearman r = 0.39, *P* < 0.01), and anxiety (SAS) (Spearman r = 0.40, *P* < 0.01).

To analyze effects of sex on dissociative and other stress-related psychopathological processes, we have used the Mann–Whitney test for independent samples and found that women have significantly higher levels of depression (BDI-II), anxiety (SAS), and traumatic symptoms (TSC-40) (*P* < 0.00007, *z* > 4.0], however, not dissociative symptoms compared with men.

## DISCUSSION

The results indicate that fatherless conditions in single parent families in boys and stepfather influence in families with girls are significantly associated with development of psychological dissociative symptoms that have clinically significant values. Other psychopathological manifestations related to mother's marital status did not reach clinical values, but may represent latent vulnerability that may manifest later in life because of specific influences of stressful experiences on brain development.^16–18^ Key results of this study represent significantly increased dissociation in young adult men whose mothers were fatherless (unmarried or divorced) and increased dissociation in women whose mothers were re-married.

In this context of differences in dissociative symptoms between women and men, results of this study show that girls and boys respond to the same mother's marital status and to limited father's presence or absence differently. Daughters of remarried mothers have pathologically increased dissociation in comparison with sons. This result is in agreement with findings suggesting that girls had more difficulties interacting with stepfathers than sons^19–21^ and some data also suggest that stepfather–daughter erotic attachment and sexual abuse is more prevalent than the abuse by biological fathers.^22–25^ In addition, mother–daughter attachment due to stepfather–daughter erotic transference (or partnership) can result to competition and conflict between mother and daughter, which may seriously affect mental disintegration and dissociative symptoms.

On the contrary, boys living with unmarried or divorced (not re-married) mothers, have clinically significant dissociation. The reason for this difference might be that, in comparison with girls, boys need a specific kind of separateness from mothers to find male identity for which they need father or father's figure.^26–34^ In addition, in the case of boys without fathers or step-fathers, there is an increased risk of compensatory erotic transference that significantly increases pathologically dependent attachment between mother and son.^35,36^ For example, recent data indicate that more than two-thirds of African American children are born to unmarried mothers,^37^ which may represent a significant factor of criminal behavior.^38,39^ These data seem to support several psychoanalytical data and interpretations regarding mental disintegration associated with pathologically dependent mother-son relationships and violence.^35,36,39^ Further research is warranted to explain to which extent psychodynamic factors play significant roles in these family processes associated with dissociation.
